# Supplementary material for: Comprehensive Genomic Analysis and Expression Profiling of Diacylglycerol Kinase (DGK) Gene Family in Soybean (Glycine max) under Abiotic Stresses
Source: Int J Mol Sci. 2019 Mar 18;20(6):1361. doi: 10.3390/ijms20061361 (PMC6470530; doi:10.3390/ijms20061361)
Supplement: Supplementary file 1 [file ijms-20-01361-s001.pdf]

Table S1. Primers used for quantitative real-time PCR analyses. F—forward; R—reverse.

Gene Name	*Primers for qRT-PCR (5′–3′)*
GmDGK1	F: CTGGCACATTCTTATGCGGATGAG
	R: CCATCCTTGTGTGCATCCAAGC
GmDGK2	F: CGGGGATCCATGTGTGACAGTTTGG
	R: GCTCTAGATTATGAAAGATTGATGGCCA
GmDGK3	F: CTTATGCGGATGAGGGCTCC
	R: CAGTTGGTCAATCAGAGTAC
GmDGK4	F: GAAAATGTCGGTGACGTGCC
	R: CGGGGTTATCAGTTTGCTGA
GmDGK5	F: GATCGTGGAGTCGTTGCG
	R: CACTCATTAGATGTTGAAGCCTC
GmDGK6	F: CATCAACAACTGGAGACACGAGC
	R: GCTTTGTGGTCGTCTTTGCTAC
GmDGK7	F: GGTCCTCGTTCGTGGAATCG
	R: CTCAGCGCGGCTAATGTAGC
GmDGK8	F: GTAAAAGGGGCAAGGGAAATG
	R: CCCCGCGGTAAGTGTGGT
GmDGK9	F: GCTACTGTGCCATTGGGGAC
	R: CTCAAGGGGTGCAATAGGGT
GmDGK10	F: CCACGGGTGACACGAACAAG
	R: CTCGGCACGGTTGATGTAGC
GmDGK11	F: TCAAACCCTTGGGCCTATAGTA
	R: ATCCAACGAACAGTCCATTGGT
GmDGK12	F: CTCTAGGACCTATTGTAGCTTCTG
	R: GGTCAGCTACATCTGTCCAACA

Table S2. The accession numbers of all the DGKs are listed. *Glycine max* (Gm), *Arabidopsis thaliana* (At), *Oryza sativa* (Os), and *Malus domestica* (Md) DGKs.

Gene Name	*Gene Locus*	*GenBank or UniProtKB Accession*	*Length (aa)*	*Molecular Mass (kDa)*	*cDNA/EST*
AtDGK1	AT5G07920	NP_196409	728	80.0	BT004148
AtDGK2	AT5G63770	NP_201182	712	79.4	AY380783
AtDGK3	AT2G18730	NP_849980	488	53.9	AY141990
AtDGK4	AT5G57690	NP_200577	487	55.5	DQ447086
AtDGK5a	AT2G20900	AAM62810	491	55.3	AY085589
AtDGK5b	AT2G20900	NP_850007	509	57.4	AC006234
AtDGK6	AT4G28130	NP_194542	466	52.5	AL035524
AtDGK7	AT4G30340	NP_567845	492	54.6	AF360174
GmDGK1	Glyma.13G302200	XP_003541921.1	430	48.62	XM_003541873.1
GmDGK2	Glyma.05G196100	XP_006580353.1	725	78.6	XM_006580290.2
GmDGK3	Glyma.12G200100	XP_014632628	480	53.7	XM_014777142
GmDGK4	Glyma.06G299200	XP_003526295	484	54.29	XM_003526247
GmDGK5	Glyma.06G223900	XP_014632628	480	53.76	XM_014777142
GmDGK6	Glyma.04G143000	XP_003552048	485	54.2	XM_003552000
GmDGK7	Glyma.13G093100	XP_003542286	480	53.54	XM_003542238
GmDGK8	Glyma.06G2549001	XP_014632201	504	56.32	XM_014776715
GmDGK9	Glyma.12G146700	XP_006592585	485	54.35	XM_006592522
GmDGK10	Glyma.17G067400	XP_014625529.3	480	53.56	XM_014770043
GmDGK11	Glyma.17G077100	XP_014625346.1	727	80.97	XM_014769860
GmDGK12	Glyma.05G022500	XP_006579516	727	80.93	XM_006579453
OsDGK1	OS04G54200	EAZ32109	541	60.3	CM000141
OsDGK2	OS08G08110	NP_001061130	502	55.8	AP008214
OsDGK3	OS02G54650	NP_001048345	488	53.9	AP005535
OsDGK4	OS12G38780	NP_001067111	705	78.7	DP000011
OsDGK5	OS03G31180	ABF96709	616	68.1	DP000009
OsDGK6	OS08G15090	BAD05689	527	57.1	AP005495
OsDGK7	OS01G57420	EEE55501	499	55.9	CM000138
OsDGK8	OS12G12260	ABG21922	663	72.8	DP000011

MdDGK1	MDP0000900186	KM099881	707	79.3	CN874967
MdDGK2	MDP0000246501	KM099882	489	54.6	CN890995
MdDGK3	MDP0000276007	EB177954	724	80.2	EB177954
MdDGK4	MDP0000139683	GO512216	502	54.5	GO512216
MdDGK5	MDP0000401076	KM099880	522	58.5	CN892391
MdDGK6	MDP0000237723	GO552958	488	54.5	GO552958
MdDGK7	MDP0000286961	KM099883	737	81.6	EB110199
MdDGK8	MDP0000171640	DR992213	538	60.2	DR992213

Table S3. Subcellular localization data found on **DeepLoc-1.0**; the uncolored cases represent the highest values for each gene in different parts of the cell. From top to bottom, the values in the table are colored differently according to clusters I, II, and III.

Genes	Nucleus	Cytoplasm	Peroxisome	Cell Membrane	Mitochondrion	Lysosome	Golgi Apparatus	Endoplasmic Reticulum	Extra Cellular	Platid
GmDGK2	0.0002	0	0.0001	0.7858	0.0008	0.0152	0.0935	0.1044	0	0
GmDGK11	0.0046	0.0004	0.0112	0.4733	0.0278	0.0728	0.1536	0.2536	0.001	0.0017
GmDGK12	0.0013	0.0003	0.0013	0.6059	0.0041	0.0395	0.1734	0.1736	0.0001	0.0006
AtDGK1	0.0003	0	0.0002	0.8015	0.0004	0.0044	0.1111	0.0821	0.0001	0
ATDGK2	0.0001	0	0	0.7834	0.0001	0.0007	0.1733	0.0422	0	0
GmDGK5	0.0781	0.5242	0.1985	0.0734	0.0589	0.0187	0.0095	0.0266	0.0055	0.0066
GmDGK6	0.2288	0.6091	0.0339	0.0066	0.0414	0.0133	0.0105	0.0124	0.0088	0.0351
GmDGK7	0.2419	0.4886	0.034	0.0586	0.092	0.0273	0.0196	0.0254	0.0053	0.0072
GmDGK10	0.463	0.3372	0.0123	0.0482	0.0688	0.0125	0.0087	0.0046	0.0067	0.0379
AtDGK3	0.4414	0.2774	0.1085	0.0666	0.0377	0.0174	0.0164	0.0151	0.0105	0.009
AtDGK4	0.3848	0.3591	0.0154	0.1309	0.008	0.043	0.0172	0.0117	0.0279	0.0021
AtDGK7	0.5091	0.3567	0.0071	0.0567	0.0048	0.0175	0.0139	0.0112	0.0222	0.0001
GmDGK1	0.227	0.4039	0.1129	0.0527	0.1044	0.0145	0.0408	0.0241	0.0071	0.0126
GmDGK3	0.2122	0.3607	0.2087	0.0677	0.0782	0.0097	0.0187	0.0174	0.0091	0.0187
GmDGK4	0.212	0.49	0.112	0.0663	0.0553	0.0173	0.014	0.0114	0.0109	0.0108
GmDGK8	0.2563	0.5964	0.0152	0.0569	0.021	0.0169	0.0117	0.0089	0.013	0.0036
GmDGK9	0.2012	0.5483	0.0758	0.0989	0.0142	0.0202	0.0154	0.0096	0.0141	0.0022
AtDGK5	0.1692	0.6145	0.0836	0.0434	0.0313	0.0186	0.0154	0.0112	0.0087	0.004
AtDGK6	0.287	0.6074	0.0077	0.0558	0.0059	0.0143	0.0076	0.0043	0.0081	0.00019

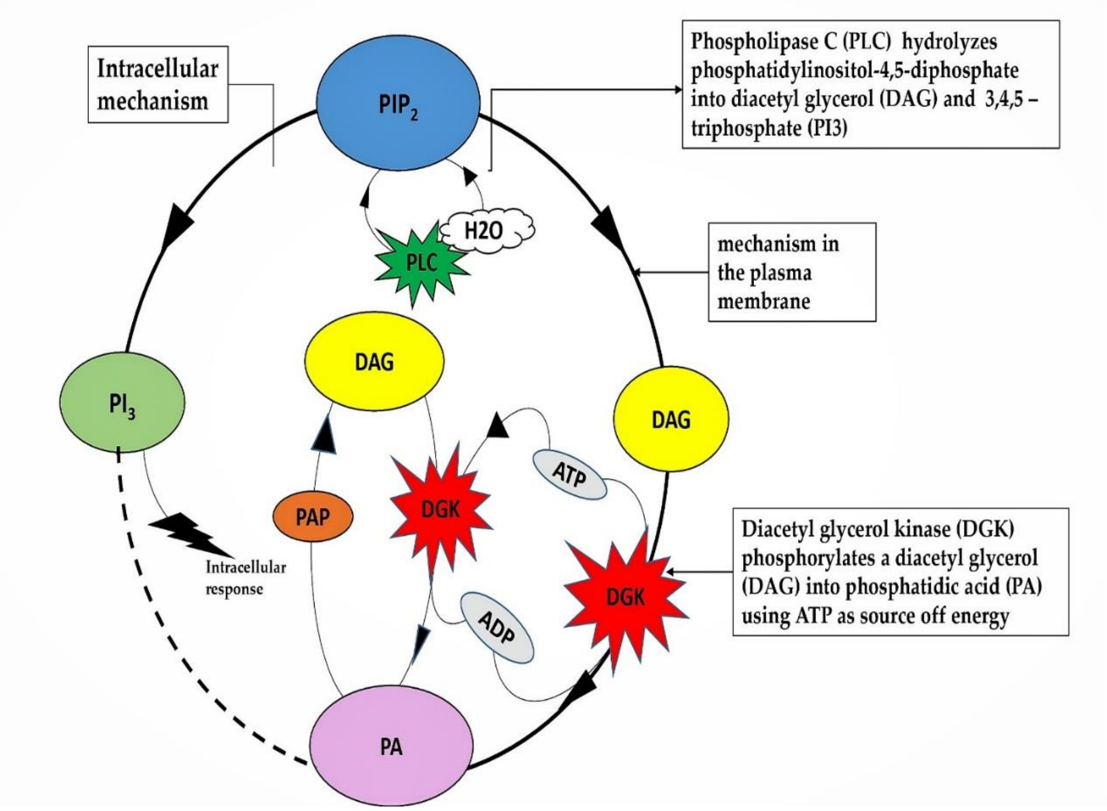

Figure S1. The general function of DGKs in eukaryotic cells, i.e., phosphate acid (PA) production via the coupled action of phospholipase C and diacetyl glycerol kinase.
